# Expression of the JAK/STAT Signaling Pathway in Bullous Pemphigoid and Dermatitis Herpetiformis

**DOI:** 10.1155/2017/6716419

**Published:** 2017-10-24

**Authors:** K. Juczynska, A. Wozniacka, E. Waszczykowska, M. Danilewicz, M. Wagrowska-Danilewicz, J. Wieczfinska, R. Pawliczak, A. Zebrowska

**Affiliations:** ^1^Department of Dermatology and Venereology, Medical University of Lodz, Lodz, Poland; ^2^Department of Pathomorphology, Medical University of Lodz, Lodz, Poland; ^3^Department of Nephropathology, Medical University of Lodz, Lodz, Poland; ^4^Department of Immunopathology, Chair of Allergy, Immunology and Dermatology, Medical University of Lodz, Lodz, Poland

## Abstract

A family of eleven proteins comprises the Janus kinases (JAK) and signal transducers and activators of transcription (STAT) signaling pathway, which enables transduction of signal from cytokine receptor to the nucleus and activation of transcription of target genes. Irregular functioning of the cascade may contribute to pathogenesis of autoimmune diseases; however, there are no reports concerning autoimmune bullous diseases yet to be published. The aim of this study was to evaluate the expression of proteins constituting the JAK/STAT signaling pathway in skin lesions and perilesional area in dermatitis herpetiformis (DH) and bullous pemphigoid (BP), as well as in the control group. Skin biopsies were collected from 21 DH patients, from 20 BP patients, and from 10 healthy volunteers. The localization and expression of selected STAT and JAK proteins were examined by immunohistochemistry and immunoblotting. We found significantly higher expression of JAK/STAT proteins in skin lesions in patients with BP and DH, in comparison to perilesional skin and the control group, which may be related to proinflammatory cytokine network and induction of inflammatory infiltrate in tissues. Our findings suggest that differences in the JAK and STAT expression may be related to distinct cytokines activating them and mediating neutrophilic and/or eosinophilic infiltrate.

## 1. Introduction

Bullous pemphigoid (BP) and dermatitis herpetiformis (DH) are both autoimmune subepidermal bullous diseases. The characteristics for the BP disease are IgG and/or C3 deposits localized along the basement membrane zone (BMZ) revealed in direct immunofluorescence examination (DIF) and circulating IgG autoantibodies present in indirect immunofluorescence (IIF) in 70% of cases [1]. The target antigens are hemidesmosomal glycoproteins present in the basement membrane of the epidermis: bullous pemphigoid antigen 1 (BPAG1) (230 kDa) and bullous pemphigoid antigen 2 (BPAG2) (180 kDa). After attachment of the antibodies to the antigens, various proinflammatory processes take place, leading to activation of neutrophils and eosinophils and release of proteolytic enzymes which contribute to blister formation [2–5]. All those processes occur with the significant participation of numerous cytokines, which elevated levels were detected in the serum and/or blister fluid of patients with BP. Moreover, levels of some of them were found correlating with activity of BP [6].

DH involves both skin and intestinal lesions. The disease is characterized by the presence of granular IgA deposits on top of the dermal papillae and in the serum IgA autoantibodies targeting endomysium and/or tissue and epidermal transglutaminase (tTG and eTG) [7, 8]. An inflammatory infiltrate, consisting of neutrophils, is considered to be crucial in DH blister formation, as neutrophils are able to release numerous proteolytic enzymes (collagenases and elastases) causing basement membrane degradation and blister formation [9].

The Janus kinases (JAK) and signal transducers and activators of transcription (STAT) are a group of proteins constituting signaling pathway present in cells of animals. Interaction between particular members of the cascade enables transmitting the signal from extracellular signaling molecules to their target genes, resulting in gene transcription.

In mammals, the STAT family is comprised of seven members (STAT1, STAT2, STAT3, STAT4, STAT5a, STAT5b, and STAT6) and there are four tyrosine kinases identified (JAK1, JAK2, JAK3, and TYK2) [10]. The cascade might be activated by numerous signaling molecules. Stimulation of the JAK/STAT pathway facilitates intercellular communication and plays significant role in cell processes such as proliferation, growth, differentiation, migration, and apoptosis. The JAK/STAT pathway is essential to normal functioning of the immune system among others [11, 12]. There has been numerous inflammatory and autoimmune diseases identified where the JAK/STAT signaling is disrupted [13–20]. However, there are no reports concerning the JAK/STAT pathway and its contribution to pathogenesis of autoimmune bullous diseases yet to be published.

The aim of this study was to evaluate the expression of proteins: JAK1, JAK2, JAK3, STAT1, STAT2, STAT3, STAT4, STAT5, and STAT6 in skin lesions and perilesional area in patients with BP and DH as well as in the control group.

## 2. Materials and Methods

### 2.1. Patients

The study included 51 persons: 20 with BP (12 women and 8 men; range 59 to 89 years; average 72,51 years) and 21 with DH (14 women and 7 men; range 19 to 62 years; average 42,46 years). All patients were at an active stage of the disease, before administration of any (systemic or topical) treatment. The control group comprised 10 healthy, unrelated volunteers, matched for sex and age. Skin samples of healthy volunteers have been taken from similar areas of those of disease's groups.

Diagnosis of BP was established based on medical history, clinical picture, and immunofluorescence findings. The histopathologic findings according to Ackerman et al. [18] in all cases were fully developed. The specimens revealed in all cases neutrophilic, eosinophilic, and lymphocytic infiltrates in dermis and in most cases (14/20) subepidermal blisters. In all patients, direct immunofluorescence tests revealed IgG/C3 linear deposits along the BMZ and in 1 M NaCl split test, deposits were observed in the epidermal side of the artificial blister or in the epidermal and dermal side of the split. Indirect immunofluorescence assay revealed circulating IgG antibodies in the serum of 14/20 patients, in titers from 1 : 80 to 320 (median 160). In the serum of 19 out of 20 patients with BP, the presence of anti-Nc16 autoantibodies was detected with ELISA (MBL, Nagoya, Japan), whereas the antibodies were absent in the control group.

DH was diagnosed based on medical history, clinical presentation, and immunofluorescence examinations as well. Biopsies were taken from skin lesions and subjected to histological examination. In all cases, the specimens revealed neutrophilic infiltrates forming papillary microabscesses. In most samples, there were unilocular, subepidermal blisters filled with fluid present, and in the remaining part of the group, small subepidermal blisters were found. All histological results were fully developed according to Ackerman et al. [18]. In all patients with DH, direct immunofluorescence assay revealed granular deposits of IgA present in skin papillae. Indirect immunofluorescence tests were positive for IgAEmA in all the patients (titer range 1 : 40–1 : 640, median 1 : 40) (Oesophagus monkey IgAEmA, Viro-Immune Labor-Diagnostika GMBH). Immunoassay (Celikey, Pharmacia & Upjohn**)** revealed antitissue transglutaminase antibodies present in the serum of 18 out of 21 patients (range 0.0–186.3 IU/ml; median 8.1 IU/ml).

Before entering the study, all the patients gave their informed written consent. The study protocol RNN/132/07/KB was approved by the Local Ethical Committee of the Medical University of Lodz.

### 2.2. Methods

The use of immunohistochemical methods versus Western blot analysis was dictated by their accessibility.

### 2.2.1. Immunohistochemistry

Immunohistochemical methods were used to evaluate expression of JAK3, STAT2, STAT4, and STAT6 in both lesional and perilesional skin and compared with healthy control skin. Paraffin-embedded tissue sections were mounted onto SuperFrost slides, deparaffinised, then treated in a solution of TRS, and transferred to distilled water. Endogenous peroxidase activity was blocked by 0,3% hydrogen peroxide in distilled water, and then sections were rinsed with Tris-buffered saline (TBS, Dako, Denmark) and incubated with primary rabbit polyclonal antibody against STAT2 (Santa Cruz Biotechnology Inc.), mouse monoclonal antibody against STAT4 (Santa Cruz Biotechnology Inc.), and primary rabbit polyclonal antibody against STAT6 (Santa Cruz Biotechnology Inc.) and incubated overnight with mouse monoclonal antibody against JAK3. Immunoreactive proteins were visualized using EnVision-horseradish peroxidase kit (Dako, Carpinteria, CA, USA) according to the instructions of the manufacturer. Visualisation was performed by incubating the sections in a solution of 3,3′-diaminobenzidine (DakoCytomation, Denmark). After washing, the sections were counterstained with hematoxylin and coverslipped. For each antibody and for each sample, a negative control was processed.

### 2.2.2. Semiquantitative Analysis

Expression was evaluated according to methodology derived from research by Tam et al. [19]. In each specimen, staining intensity of JAK3, STAT2, STAT4, and STAT6 was recorded semiquantitatively by two independent observers in 7–9 high-power fields using in each field a weighted histoscore method according to Kirkegaard et al. 2006, also known as the H score system [20]. The immunoexpression score was calculated as follows: (1 × % cells staining weakly positive) + (2 × % cells staining moderately positive) + (3 × % cells staining strongly positive). The mean score for each specimen was calculated by averaging grades assigned by the two authors and approximating the arithmetical mean to the nearest unity. All values were expressed as the mean ± SD (standard deviation).

### 2.2.3. Western Blot

Western blot method was used to evaluate the expression of JAK1, JAK2, STAT1, STAT3, and STAT5 in skin lesions of DH and BP groups (and not perilesional skin) and compared with healthy control group. STAT5 antibody recognized both STAT5a and STAT5b.

Total proteins from frozen skin samples from BP and DH patients and healthy controls were extracted in RIPA protein extraction buffer, supplemented with protease inhibitor cocktail (Sigma-Aldrich, St. Louis, MO, USA). The lysate was centrifuged, and the pellet was discarded. Protein concentrations were determined by the BCA Protein Assay Kit (Pierce Thermo Scientific, USA) according to manufacturer's instructions.

The membrane was blocked with nonfat milk in TBST and then incubated with the mouse primary antibodies (Santa Cruz Biotechnology, Dallas, USA). Afterwards, the membrane was incubated with secondary goat anti-mouse IgG polyclonal antibodies conjugated with alkaline phosphatase (Santa Cruz Biotechnology, Dallas, USA). The bands were developed using BCIP/NBT Alkaline Phosphatase Substrate (Merck Millipore, Darmstadt, Germany) and analyzed using the ImageJ 1.34s software (Wayne Rasband, National Institutes of Health, Bethesda, MD), which allowed for image analysis of densitometry expressed as % optical density (OD) over the background. Obtained results were expressed as the mean ± SD.

### 2.2.4. Statistical Methods

The results were presented as the mean ± SEM. The data were analyzed using Statistica (v. 10.0; StatSoft, Tulsa, OK, USA). The distribution of the data and the equality of variances were checked by Levene's test. Differences between groups were tested using ANOVA (WB) and unpaired Student's *t*-test (immunohistochemistry), and the Mann–Whitney *U* test was used where appropriate. The level of significance was defined where *p* < 0.05.

## 3. Results

### 3.1. WB

**Figure 1:**
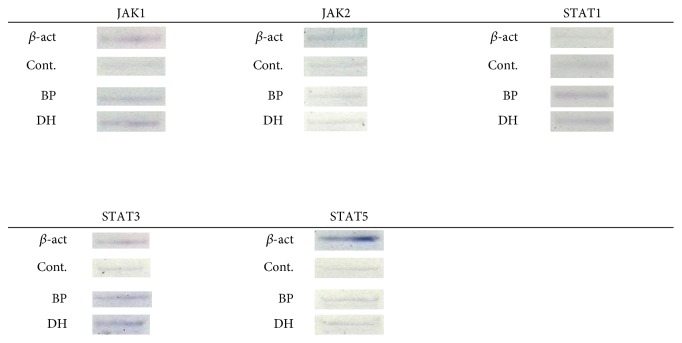
Expression of JAK1, JAK2, STAT1, STAT3, and STAT5 in epidermis, evaluated with Western blot. *β*-act—positive control. Cont.—control group, normal skin. BP—skin lesions, bullous pemphigoid. DH—skin lesions, dermatitis herpetiformis.

Expression of JAK1 was evaluated as one of the highest in patients with DH (140.27 ± 0.15) and BP (141.10 ± 1.50) and the control group (141.10 ± 0.51) (Figure 1). However, there were no statistical differences between expression of the protein in the control group and DH patients (*p* > 0.05) and between the control group and BP patients. No statistical difference in JAK1 expression was found between BP and DH patients as well.

The intensity of JAK2 expression was higher in BP patients (133.48 ± 0.84) as compared to patients with DH (130.21 ± 0.96; *p* < 0.05) and the control group (130.42 ± 1.65; *p* < 0.05). There was no statistical difference (*p* > 0.05) between JAK2 expression in DH lesions and healthy skin.

The expression of STAT1 was evaluated higher in BP patients (145.83 ± 0.25) and DH patients (143.85 ± 3.09) as compared to the control group (136.28 ± 2.84; *p* < 0.05). There was no significant difference between STAT1 expression in DH and BP patients.

The intensity of STAT3 expression was higher in BP patients (138.39 ± 0.84) and DH patients (141.2 ± 0.05) as compared to the control group (121.63 ± 1.75; *p* < 0.05). There was also statistical significance between expression on STAT3 in DH and BP patients, in favor of DH skin lesions.

**Figure 2:**
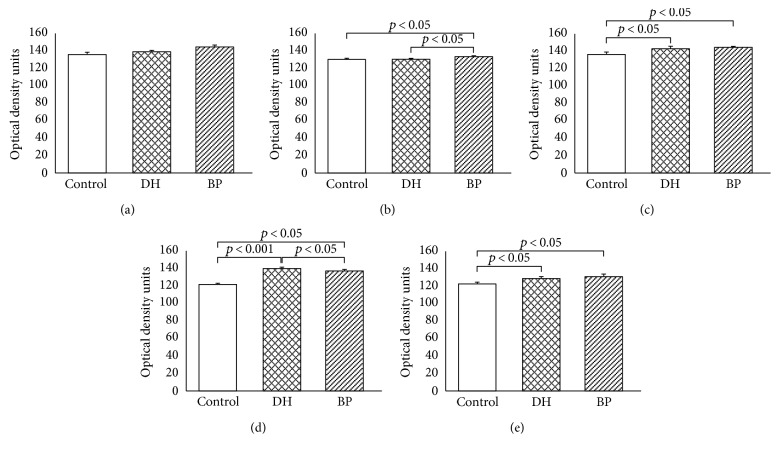
Morphometric analysis of JAK1 (a), JAK2 (b), STAT1 (c), STAT3 (d), and STAT5 (e) immunoexpression in keratinocytes, evaluated with Western blot. The results are presented as % optical density over the background (the mean ± SD). Control—normal skin. DH—dermatitis herpetiformis, skin lesions. BP—bullous pemhigoid, skin lesions. The level of significance is defined where *p* < 0.05. JAK1: C versus DH (NS), C versus BP (NS), and DH versus BP (NS). JAK2: C versus DH (NS), C versus BP (*p* < 0.05), and BP versus DH (*p* < 0.05). STAT1: C versus DH (*p* < 0.05), C versus BP (*p* < 0.05), and DH versus BP (NS). STAT3: C versus DH (*p* < 0.05), C versus BP (*p* < 0.05), and DH versus BP (*p* < 0.05). STAT5: C versus DH (*p* < 0.05), C versus BP (*p* < 0.05), and DH versus BP (NS).

Expression of STAT5 protein was significantly higher in BP patients (131.37 ± 2.55) and DH patients (129.34 ± 1.37) as compared to the control group (123.48 ± 1.13; *p* < 0.05). There was no statistical difference in expression of STAT5 in DH and BP patients (Figures 1 and 2).

### 3.2. Immunohistochemistry

**Figure 3:**
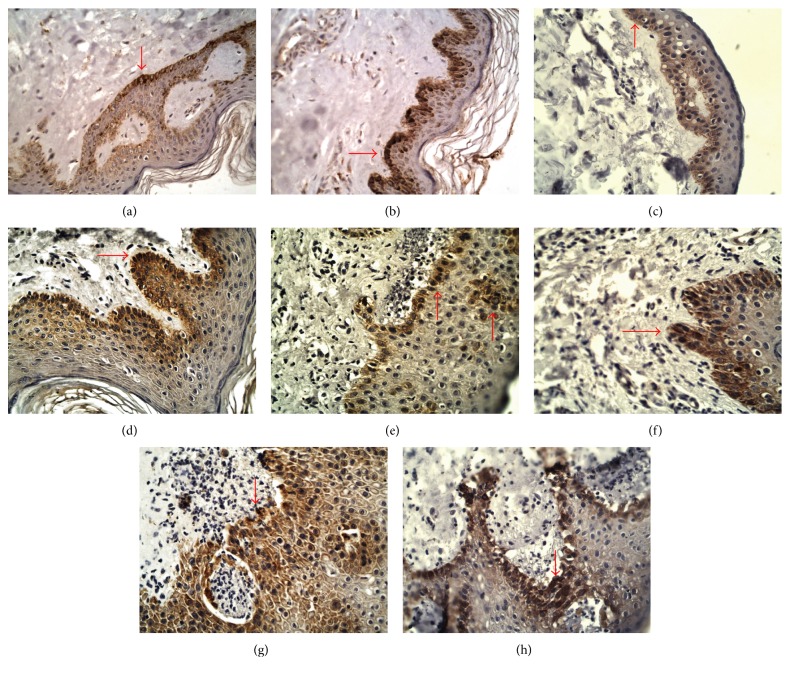
Immunoexpression of JAK/STAT proteins in epidermis, DH, 400x, immunohistochemistry. Immunoexpression of JAK3 in epidermis (a) perilesional skin 14.38 ± 3.61 and (e) skin lesions 18.89 ± 4.67, *p* > 0.05; immunoexpression of STAT2 in epidermis (b) perilesional skin 15.79 ± 2.06 and (f) skin lesions 17.15 ± 2.81. NS; immunoexpression of STAT4 in epidermis (c) perilesional skin 24.10 ± 3.40 and (g) skin lesions 29.08 ± 4.38, *p* < 0.05; immunoexpression of STAT6 in epidermis (d) perilesional skin 18.21 ± 3.49 and (h) skin lesions 27.85 ± 4.68, *p* < 0.05.

**Figure 4:**
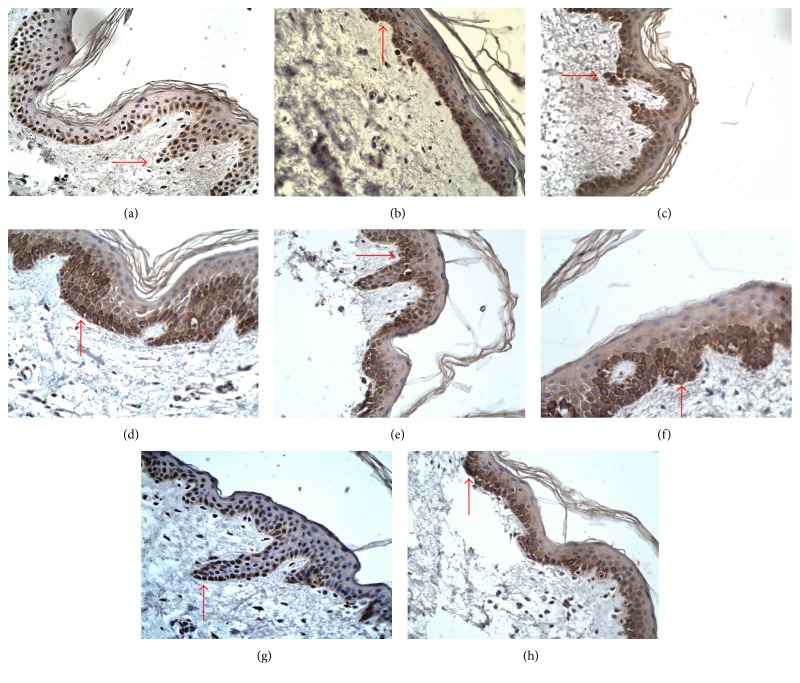
Immunoexpression of JAK/STAT proteins in epidermis, BP, 400x, immunohistochemistry. Immunoexpression of JAK3 in epidermis (a) perilesional skin 12.54 ± 2.99 and (e) skin lesions 18.19 ± 5.58, *p* > 0.05; immunoexpression of STAT2 in epidermis (b) perilesional skin 14.01 ± 2.38 and (f) skin lesions 17.32 ± 2.69, *p* > 0.05; immunoexpression of STAT4 in epidermis (c) perilesional skin 21.05 ± 2.91 and (g) skin lesions 25.13 ± 3.56, NS; immunoexpression of STAT6 in epidermis (d) perilesional skin 17.46 ± 2.41 and (h) skin lesions 26.09 ± 4.45, NS.

**Figure 5:**
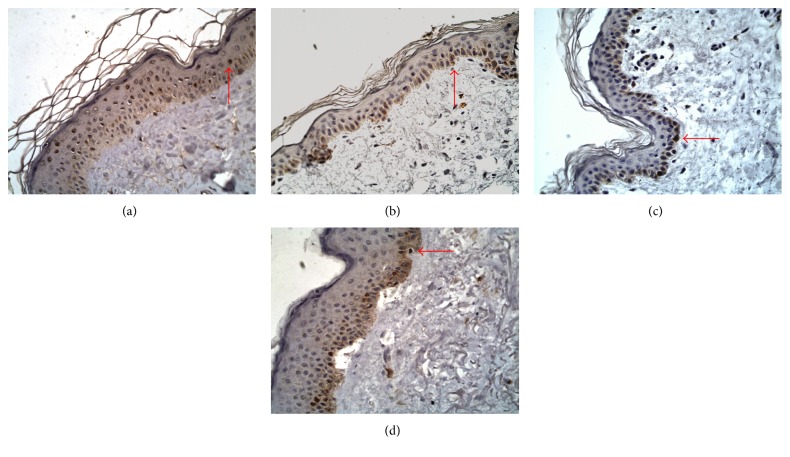
Immunoexpression of JAK/STAT proteins in epidermis, normal skin, 400x, immunohistochemistry. (a) Immunoexpression of JAK3 in epidermis, normal skin, 10.73 ± 3.36. (b) Immunoexpression of STAT2 in epidermis, normal skin, 11.06 ± 5.34. (c) Immunoexpression of STAT4 in epidermis, normal skin, 18.59 ± 3.01. (d) Immunoexpression of STAT6 in epidermis, normal skin, 11.56 ± 2.84.

In the healthy skin samples, expression of JAK3 was found throughout the epidermis with the horny layer being strongly stained by the antibody against JAK3. Immunoreactivity of STAT2, STAT4, and STAT6 antibodies were more strongly detected in the granular layer than in lower layers of the epidermis. The horny cell layers were not stained with the antibodies against STAT2, STAT4, and STAT6 (Figures 3, 4, and 5).

Expression of JAK3 was higher in BP skin lesions (18.19 ± 5.58) and DH skin lesions (18.89 ± 4.67) in comparison with the control group (10.73 ± 3.36; *p* < 0.05). There were no statistically significant differences between the control group and BP perilesional skin (12.54 ± 2.99; *p* > 0.05) and DH perilesional skin (14.38 ± 3.61; *p* > 0.05). However, there were statistically significant differences between expression of JAK3 in skin lesions and perilesional area in patients with BP (*p* < 0.05) and DH (*p* < 0.05). There was no significant difference (*p* > 0.05) between JAK3 expression in BP skin lesions and DH skin lesions. There was also no statistical difference (*p* > 0.05) between expression of JAK3 in BP perilesional skin and DH perilesional skin.

Expression of STAT2 was higher in BP patients' skin lesions (17.32 ± 2.69) and DH patients' skin lesions (17.15 ± 2.81) than in the control group (11.06 ± 5.34; *p* < 0.05). There was no significant difference between STAT2 expression in the control group and BP perilesional area (14.01 ± 2.38; *p* > 0.05), but there was significant difference between STAT2 expression in the control group and DH perilesional skin (15.79 ± 2.06; *p* < 0.05). However, there was no statistically significant difference between STAT2 expression in BP skin lesions and DH skin lesions (*p* > 0.05) and between BP perilesional skin and DH perilesional skin (*p* > 0.05). There was a statistical difference evaluated between BP skin lesions and perilesional area (*p* < 0.05), but there was no statistical significance between expression of STAT2 in DH skin lesions and DH perilesional area (*p* > 0.05).

Expression of STAT4 was higher in DH skin lesions (29.08 ± 4.38) and BP lesions (25.13 ± 3.56) as compared to the control group (18.59 ± 3.01; *p* < 0.05). There was also significant difference between expression of STAT4 in the control group and DH perilesional skin (24.10 ± 3.40; *p* < 0.05), but there was no significant difference between STAT4 expression in healthy skin and BP perilesional area (21.05 ± 2.91). There was a statistical significance between STAT4 expression in skin lesions of BP patients and DH patients (*p* < 0.05). There were also differences between expression of STAT4 in skin lesions and perilesional area in patients with BP (*p* < 0.05) as well as in patients with DH (*p* < 0.05).

**Figure 6:**
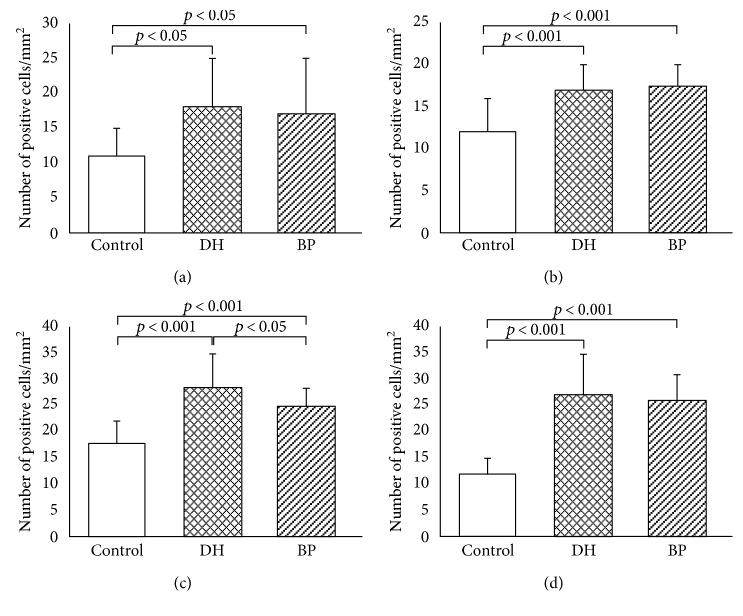
Morphometric analysis of JAK3 (a), STAT2 (b), STAT4 (c), and STAT6 (d) immunoexpression in keratinocytes, evaluated with immunohistochemistry. The results of semiquantitative analysis are expressed as the mean ± SD. Control—normal skin. DH—dermatitis herpetiformis, skin lesions. BP—bullous pemphigoid, skin lesions. The level of significance is defined where *p* < 0.05. JAK3: C versus DH (*p* < 0.05), C versus BP (*p* < 0.05), and DH versus BP (NS). STAT2: C versus DH (*p* < 0.05), C versus BP (*p* < 0.05), and BP versus DH (NS). STAT4: C versus DH (*p* < 0.05), C versus BP (*p* < 0.05), and DH versus BP (*p* < 0.05). STAT6: C versus DH (*p* < 0.05), C versus BP (*p* < 0.05), and DH versus BP (NS).

The medium intensity of STAT6 expression was higher in BP skin lesions (26.09 ± 4.45) and DH skin lesions (27.85 ± 4.68) as compared to the control group (11.56 ± 2.84; *p* < 0.05). There were also significant differences between STAT6 expression in the control group and perilesional BP skin (17.46 ± 2.41; *p* < 0.05), as well as between healthy skin and DH perilesional skin (18.21 ± 3.49; *p* < 0.05). There were statistical differences (*p* < 0.05) between STAT6 medium expression in lesional and perilesional area in both patients with BP and DH (Figure 6). There were also statistical differences between STAT6 expression in BP skin lesions and BP perilesional skin (*p* < 0.05) and between DH skin lesions and DH perilesional skin (*p* < 0.05).

## 4. Discussion

Nishio et al. [21] have studied immunolocalisation of the JAK/STAT pathway in human epidermis. Using immunohistochemical methods, they noted higher expression of JAK2, JAK3, STAT1, and STAT5 in the horny cell layer and abundant expression of JAK3, TYK2, STAT2, STAT3, STAT4, and STAT6 in the granular layer of the epidermis. Our immunohistochemical findings in the control group confirm differential expression and immunolocalisation of JAK3, STAT2, STAT4, and STAT6 in healthy human epidermis and are consistent with the results by Nishio et al. It is suggested that elements of the pathway may play an important role in keratinocyte differentiation [22, 23]. This might suggest that the elementary level of expression of JAK/STAT proteins is necessary to normal functioning of the epidermis.

The JAK/STAT signaling pathway might be activated by numerous signaling cytokines, growth factors and hormones, such as interferon-*α*/*β*/*γ* (IFN-*α*/*β*/*γ*), IL-2, IL-4, IL-6, IL-7, IL-9, IL-10, IL-12, IL-15, IL-19, IL-20, IL-21, IL-22, IL-23, erythropoietin (Epo), growth hormone (GH), prolactin (PRL), thrombopoietin (TPO), granulocyte colony-stimulating factor (G-CSF), epidermal growth factor (EGF), platelet-derived growth factor (PDGF), and leptin [9, 24]. After attachment of the signaling molecule to its transmembrane receptor, activation of the associated with the cytoplasmic domain of the receptor JAK takes place. When activated, JAKs phosphorylate cytokine receptors, which enables STAT monomers present in cytoplasm to bind to the complex and form homo- and heterodimers due to tyrosine phosphorylation. Then, activated STATs translocate to the cell nucleus and bind to DNA, enabling transcription of target genes [25].

In the pathomechanism of BP, degranulation of mast cells is considered significant in the inflammatory cascade and blister formation. This process may be induced by C3a and C5a components of the complement, activated after attachment of the antibodies to the BP antigens [4] or by IgE BP 180 antibodies [26]. There are numerous mediators released from mast cells, such as TNF-*α*, platelet-activating factor (PAF), metalloproteinase, leukotrienes, histamine, and other cytokines (IL-1, IL-2, IL-5, and IL-6) resulting in chemoattraction of neutrophils and eosinophils along the BMZ and their activation [2–5]. A variety of proteolytic enzymes produced by such activated neutrophils and eosinophils contributes to blister formation.

In all mentioned mechanisms, various signaling molecules take part. It is reported that patients with BP show an increased expression of numerous cytokines and chemokines, and many of them are involved in chemoattracting and activating eosinophils and neutrophils, which is thought to be crucial in blister formation. Recent literature data indicates that elevated levels of IL-1, IL-2, IL-4, IL-5, IL-6, IL-8, IL-10, IL-15, IL-16, IL-17, tumor necrosis factor *α* (TNF-*α*), and chemokine ligand 18 (CCL-18) were found in the serum and/or blister fluid of patients with BP [22, 23, 27–36]. As the JAK/STAT pathway is one of the most important signaling pathways for cytokines and growth factors, differences between the expression levels of particular JAK and STAT proteins in BP and DH may be related to distinct cytokines activating them and mediating eosinophilic and/or neutrophilic infiltrate.

STAT4 and STAT6 are considered as being remarkably involved in inflammatory processes, as they are activated by proinflammatory cytokines, such as IL-12 and IFN-*γ* (both activate STAT4) and IL-4 and IL-13 (activating STAT6) [37, 38]. It is reported that JAK2/STAT4, as a IL-12 signaling components, are critical for Th1 cell differentiation, and JAK1/3/STAT6, as a IL-4 signaling pathway, are essential for Th2 differentiation [39]. Our results suggest that both STAT4 and STAT6 may contribute to BP pathogenesis, probably due to their contribution to Th1 and Th2 immune responses. It is suggested in literature that in addition to humoral immune response, there are also autoimmunity mechanisms in BP concerning cellular immune response, involving autoreactive T and B cells [4]. Recent reports show that there are many cytokines released by autoreactive T cells (Th1/Th2-mixed cytokine profile), which elevated levels were detected in the serum and/or blister fluid of patients with BP, especially IL-4, IL-5, and IL-2 [6].

The results of our research suggest also that STAT4 and STAT6 may contribute to DH pathogenesis. Among proteins evaluated with immunohistochemichal methods in DH skin lesions, the expression of STAT4 is the highest. The contribution of STAT4 and STAT6 to DH pathogenesis needs to be further examined. The probable mechanism is dependent on IL-12 signaling and participation of STAT4/STAT6 in Th1/Th2 immune response mechanisms.

Despite the role of STAT3 in promoting oncogenesis, STAT3 is able to transduce and transmit signal initiated by proinflammatory cytokines: IL-6 (to a greater extent than STAT1), IL-21, and IL-23 [40–42]. STAT3 is also involved in promoting production of IL-8 by regulating its transcription [42, 43]. Reports concerning transmitting IL-17 signal by STAT3 are equivocal [32, 33, 44–47]. However, numerous reports show that STAT3 mediates IL-23- and IL-6-dependent signals and critically influences the Th17 cell differentiation and hence indirectly promotes IL-17A transcription [48]. STAT3 is also a part of JAK1/STAT3-STAT5b pathway, mediating TNF-*α* biological action, estimated essential in the pathogenesis of BP and DH [49]. There is an alternative inflammatory mechanism in BP reported, the one mediated by IL-17. This cytokine is able to induce Th2 cell activation, directly recruit neutrophils and eosinophils, and produce proinflammatory cytokines (TNF-*α*, IL-1, IL-6, IL-8, granulocyte macrophage colony-stimulating factor (GM-CSF), and metalloproteinases) [50]. This optional inflammatory pathway mediated by IL-17 is not induced by antibodies and evades the complement activation and mass cell degranulation, which may lead directly to subepidermal blistering.

In our research, the expression of STAT3 in BP skin lesions suggests participation of the protein in pathogenesis of bullous pemphigoid. This may relate to contribution of IL-6, TNF-*α*, IL-8, and IL-17 to pathogenesis of the disease. It is reported that serum levels of TNF-*α*, IL-6, and IL-8 among others were found correlating with intensity of the BP disease, which underlines their pathological relevance [29–31, 34]. We suggest that expression of STAT3 may correlate with intensity of the BP disease; however, this hypothesis requires further studies.

The neutrophilic infiltrate is thought to play crucial role in the patomechanism of blister formation in DH. Such an influx may be initiated by complement components activated by IgA binding to target receptors; however recently, the important role of IL-8, GM-CSF, and TNF-*α* in inflammation cascade is underlined [51–55]. Evidence shows that in DH patients, expression of IL-8 in both serum and basal layer of the epidermis is increased. [52, 53]. This has significant meaning, as IL-8 is able to chemoattract and activate neutrophils. Apart from IL-8, it is reported that in the dermoepidermal junction of DH skin lesions, an increased expression of GM-CSF is present, which is able to activate neutrophils and induce IgA receptors. The other cytokines relevant to immune mechanisms and development of an inflammatory influx which increased expression was confirmed in DH patients are TNF-*α* and IL-17 [36, 55]. STAT3 expression results suggest that it may relate to pathogenesis of DH, mediating signal initiated by IL-6 and TNF-*α* (STAT1/STAT5) and contributing to IL-8 and IL-17 transcription. We suggest that STAT3 expression, significantly higher in DH lesions than in BP lesions, relates to crucial role that IL-8 plays in subepidermal blister formation in DH.

As reported, STAT1 is involved in transmitting signal initiated by IL-6-type cytokines (including IL-6, IL-11, oncostatin M (OSM), leukaemia inhibitory factor (LIF), cardiotrophin-11, ciliary neurotrophic factor, and IL-5 (JAK2/STAT1 pathway) among others [40, 56]. Interferons use JAK/STAT pathway to transmit their signals as well. Interferons *α* and *β* activate JAK1 and TYK-2 kinases and subsequently STAT1 and STAT2 proteins, while IFN-*γ* uses JAK1 and JAK2 kinases and STAT1 homodimers in signal transmitting [57, 58]. In the current research, the expression of STAT1 suggests participation of STAT1 in pathogenesis of both blistering diseases. It is also suggested that this participation may relate to IL-6, interferons *α* and *β*, and IL-5 signaling (BP).

The expression of STAT2, significantly higher in BP and DH skin lesions than in healthy skin, suggests participation of STAT2 and cytokines activating it (interferons *α* and *β*) in pathogenesis of both diseases. Moreover, the expression of STAT2 in DH skin lesions was evaluated similar to the expression of STAT2 in perilesional skin, and it was higher in healthy skin. It is reported that IgA deposits are present on top of the dermal papillae not only in skin lesions but also in perilesional skin as well. They can be also present in remission phase [59]. It is concluded that inflammatory processes take place in perilesional area as well. The results of our research suggest that STAT2 may be involved in proinflammatory reactions taking place in DH perilesional area.

Similarly to STAT3, aberrant activation of STAT5 has been implicated in the pathogenesis of hematologic and solid-organ malignancies [44, 60]. Additionally, STAT5 is reported to transmit signal initiated by PRL, IL-3 family (IL-3, IL-5, and GM-CSF), IL-2 family (IL-2, IL-7, TSLP, IL-9, IL-15, and IL-21), GH, EPO, and TPO [25, 61, 62]. The results of our research suggest that STAT5 may contribute to the pathogenesis of both BP and DH; however, the estimated STAT5 expression is the lowest one among analyzed with WB proteins. The potential contribution to pathogenesis of bullous diseases may be related to Th1 immune response and IL-5 signaling (BP); however, it requires further studies.

JAK3 is the only JAK protein which expression is higher in DH skin lesions than in the healthy control group. JAK3 is involved with receptors with common *γ* chain (IL-2, IL-4, IL-7, IL-9, IL-15, and IL-21), crucial to lymphocyte maturation and function [63]. It is suggested that JAK3 contributes to the pathogenesis of DH, probably by mediating response to IL-4 and participation in Th17 differentiation (JAK3/STAT3 pathway).

The results of the current research suggest that both JAK2 and JAK3 may contribute to the pathogenesis of the BP. JAK2 acts on receptors of IFN-*γ* and IL-5 among others [46, 63]. We suggest that JAK2 expression may relate to IL-5 and IFN-*γ* signaling, while JAK3 expression may be dependent on IL-4 signaling and Th17 immune response.

Estimated expression of JAK1, similar in every examined sample, suggests that this protein does not participate in pathogenesis of BP and DH. It is accepted that JAK1 is generally associated with IFNs, IL-6 and IL-10 family receptors, and receptors with common *γ* chain [46, 63]. The results of our research suggest that activity of JAK1 is necessary to normal functioning of healthy skin and it helps to keep immunological homeostasis. The other hypothesis concerning JAK1 is that proinflammatory signaling via JAK1 may be present in autoimmune subepidermal blistering diseases in an active phase of the diseases, however at low level.

Although BP and DH share elevated expression of some cytokines (TNF-*α*, IL-6, IL-8, and IL-17), there are also significant differences in cytokine pattern involved in pathogenesis of both diseases. Eotaxin, eosinophilic cationic protein (ECP), and especially IL-5, known as activator of eosinophils, were found in BP blister fluid only [32, 64]. Differences between the presence and expression of various cytokines in BP and DH patients are thought to relate do different infiltrates in both diseases—neutrophilic in DH—and an infiltrate formed by eosinophils and neutrophils in BP. In our research, skin lesions of both diseases differ with statistical significance in the expression levels of JAK2, STAT3, and STAT4. In DH skin lesions, higher expression of STAT3 and STAT4 was observed, which may contribute to crucial role of IL-6, TNF-*α*, IL-8, IL-17, and IL-12 in pathogenesis of DH, which is consistent with findings concerning cytokine levels in DH [65–67]. The expression of JAK2 in BP skin lesions is more significant than in DH and may contribute to essential role of IL-5 in pathogenesis of eosinophilic component of inflammatory infiltrate in BP, as in eosinophils IL-5 transmit signal via JAK2-STAT1/STAT5 and thus regulate transcription of genes involved in cell proliferation and effector functions [68]. Moreover, the pathological relevance of IL-5 is underlined by the correlation between IL-5 level in blister fluid and intensity of the BP disease [29–31, 34].

It is important to underline that relation between cytokine levels and JAK/STAT expression must not always be directly proportional, as regulation of the JAK/STAT pathway and the use of alternative signaling pathways may contribute to differentiation in expression of JAK and STAT proteins. Negative regulation of the pathway includes inhibition of activity of JAKs and STATs, among others. Suppressor of cytokine signaling (SOCS) proteins are able to bind to JAK proteins and inactivate their kinase activity while PIAS (protein inhibitors of activated STATs) have the function of preventing STATs from specific DNA recognition by direct binding to activated STAT dimers [69, 70]. Moreover, the activity of the JAK/STAT pathway might be influenced by chemical modifications of STATs, like acetylation, methylation, sumoylation, and phosphorylation [71].

The JAK/STAT pathway is crucial, however not the only cytokine signaling cascade present in cells. Cytokines such as IL-1, IL-8, and M-CSF may use alternative to JAK/STAT ways of signaling [72]; hence, elevated serum/blister fluid levels of some cytokines may not always be related to increased activation of JAK/STAT pathway. The other example is IL-5, which may not only signal through JAK/STAT pathway but may also use mitogen-activated protein kinases (MAPK) as well [68]. As explicit above, apart from transmitting signal, the JAK/STAT pathway (e.g., STAT3 protein) may be involved in transcription of proinflammatory cytokines important in DH and BP patomechanisms, such as IL-8 and IL-17 which can additionally implicate differences in JAK and STAT levels.

Expression of JAK/STAT proteins in BP and DH and their suggested role in pathogenesis of both diseases creates new potential therapeutic targets for the treatment of subepidermal blistering diseases. JAK inhibitors have shown beneficial results in skin diseases, like psoriasis and allergic dermatitis [46]. First JAK inhibitors (Jakinibs) to be tested in humans include Tofacitinib (anti-JAK3, anti-JAK1, and anti-JAK2), Ruxolitinib (anti-JAK1 and anti-JAK2), and Baricitinib (anti-JAK1 and anti-JAK2). Those therapeutic agents are able to inhibit a relatively broad spectrum of cytokines and chemokines, which implicates their efficacy and adverse effects; hence, it remains attractive to more selectively target JAK proteins [46, 73, 74]. At present, there are over twenty Jakinibs being tested in various diseases, most of them targeting a single JAK (second-generation Jakinibs) [46].

Given the expression pattern of JAKs, it is suggested that patients with DH may benefit especially from targeted JAK3 inhibition. Inhibiting JAK3/STAT3 pathway may result in decreased IL-8 transcription, decreased Th17 cell differentiation, and hence impaired IL-17 production, as well as defective IL-6 signaling. Similar effects are observed in murine models after administrating Tofacitinib [75]. In BP patients, inhibiting JAK2 and JAK3 signaling could be promising, potentially resulting in impaired IL-4, IL-5, and IFN-*γ* signaling and disrupted IL-17 production. The promising perspective might be the use of Ruxolitinib in both BP and DH, as Ruxolitinib inhibits JAK1 and JAK2 pathways through blocking STAT3 phosphorylation, resulting in the suppression of Th17 cell differentiation, essential in both DH and BP pathogenesis [75].

The presented study is the first one exploring the expression of JAK/STAT proteins in bullous diseases yet to be published and a sparse one, presenting the expression of the entity of the JAK/STAT proteins. It would be of great importance to compare our findings with results obtained in a comparable research. Undoubtedly, the examined issue brings prospects for future studies. The comparison of particular JAK/STAT expressions and cytokine levels in serum and blister fluid could give more detailed data on pathogenic role of examined proteins and cytokines in BP and DH. The other interesting perspective would be to investigate the correlation between JAK/STAT expression and clinical features, like frequency of itch or BPDAI. Conclusions of such researches could give more comprehensive understanding of pathogenesis of bullous diseases.

The use of different analytical methods (immunohistochemical versus Western blot analysis) was dictated by their availability. Our choice was associated with the urge to evaluate as many proteins of this pathway as possible. We are aware that it would be more appropriate to use a homogeneous method, as a methodology unification could facilitate comparison between expressions of particular proteins.

## 5. Conclusions

The current study is the first one exploring the expression of JAK/STAT proteins in BP and DH to be published. The obtained results demonstrate increased expression of JAK/STAT proteins in skin lesions in patients with BP and DH, in comparison to perilesional skin and control group, which may be related to proinflammatory cytokine network and induction of inflammatory infiltrate in tissues. This can also make a contribution to pathogenesis of BP and DH skin lesions. Our findings suggest that differences between the expression of particular JAK and STAT proteins in BP and DH may be related to distinct cytokines activating them and mediating neutrophilic and/or eosinophilic infiltrate. This can make a contribution to pathogenesis of BP and DH skin lesions. However, this issue requires further studies, as a target for new therapeutic agents (anti-JAK and anti-STAT) as well.
